# Recent research progress of photoacoustic endoscopy in the digestive system

**DOI:** 10.1097/eus.0000000000000127

**Published:** 2025-07-03

**Authors:** Kai Zhang, Nan Ge, Lufan Shen, Fei Yang, Jianjun Qiu, Jintao Guo, Kaixuan Wang, Sheng Wang, Fan Yang, Shiyun Sheng, Tianan Jiang, Zhendong Jin, Siyu Sun

**Affiliations:** 1 Department of Gastroenterology, Endoscopic Center, Shengjing Hospital of China Medical University, Shenyang, Liaoning Province, China; 2 Engineering Research Center of Ministry of Education for Minimally Invasive Gastrointestinal Endoscopic Techniques, Shengjing Hospital of China Medical University, Shenyang, Liaoning Province, China; 3 Research Institute, SonoScape Medical Corporation, Shenzhen, Guangdong Province, China; 4 Department of Gastroenterology, Changhai Hospital, Naval Medical University, Shanghai, China; 5 Department of Ultrasound Medicine, The First Affiliated Hospital, Zhejiang University School of Medicine, Hangzhou, Zhejiang Province, China.

## Introduction

Photoacoustic imaging (PAI) was developed in the 1990s, based on Alexander Graham Bell’s discovery of the photoacoustic effect.^[1]^ The principles of PAI state that tissues exposed to brief light pulses absorb and transform energy into heat, increasing the local temperature and leading to thermal expansion and generation of ultrasonic waves. Such ultrasonic waves (i.e., photoacoustic signals) are detected using ultrasonic detectors positioned outside the tissue, with the creation of a corresponding photoacoustic image.^[1]^

The benefits of PAI include high spatial resolution, strong image contrast, and deep imaging depth.^[2]^ PAI can obtain information on hemoglobin, lymphatics, lipid composition, and blood oxygen metabolism.^[3–7]^ Thus, its use has significantly improved the sensitivity and specificity of cancer diagnosis.^[8–10]^

Recently, significant advancements have been made in photoacoustic tomography (PAT), which uses the photoacoustic effect to generate 3-dimensional (3D) and cross-sectional images.^[11,12]^ In addition to being speckle-free,^[13]^ PAT obtains deep, high spatial resolution images by detecting ultrasonic waves, as ultrasonic scattering is approximately 2 to 3 orders of magnitude lower than optical scattering,^[12,14,15]^ and acoustic diffraction is 2 to 3 orders of magnitude lower than the radiofrequency equivalent. PAT comprises 3 main imaging modalities: photoacoustic endoscopy (PAE), photoacoustic computed tomography,^[16,17]^ and photoacoustic microscopy (PAM).^[18,19]^ Moreover, PAT can be integrated with other imaging methods such as optical coherence tomography,^[20–22]^ confocal microscopy,^[23]^ magnetic resonance imaging,^[24]^ and ultrasound (US) imaging.^[5]^

The PAE system integrates an optical fiber, transducer, and lens into the endoscope probe and performs endoscopic imaging using a miniature probe for both light delivery and acoustic detection.^[25]^

The current summative studies of PAE did not specifically target digestive tract diseases. Hence, we aimed to focus on the development and potential use of PAE devices for digestion evaluation.

## Clinical demands for PAE

Although visible-light endoscopy is frequently used for the visualization of the intestinal wall to diagnose various disorders, gastroenterologists have been unable to identify early epithelial dysplastic alterations that may lead to cancer development.^[26]^

In addition to endoscopy, EUS, which was first reported in 1980, has rapidly developed in recent decades,^[27–30]^ allowing the acquisition of high-resolution, large-penetration-depth images of the gastrointestinal system and adjacent tissues.^[31,32]^ However, the potential of soft-tissue imaging to provide physiologically precise functional information is constrained by sonic-based picture contrast. In contrast, PAE is ideal for soft tissue imaging due to its excellent functional optical contrast and spatial resolution. Moreover, as PAE and EUS utilize the same transducer, combining the 2 modalities to achieve multimodality PAE is simple, enabling the assessment of the digestive tract structure and depth of tumor invasion, as well as the microvascular morphology and blood oxygen saturation within a few millimeters of the superficial surface of the intestinal wall. Such combined technology provides doctors with multiscale, multiparametric imaging, which aids in the early detection and treatment of diseases, in addition to providing new concepts and techniques for the high-resolution imaging of lesion depth and targeted detection of tumor neovascularization.

## PAE development

In 2009, Yang et al. developed a unique PAE technique using a miniature imaging probe, which allowed researchers to obtain *ex vivo* or *in situ* images of the digestive system of rats^[33]^; this was the first report on PAE [Table 1].

**Table 1:** Development of PAE

Year	Research Group	Modalities	Probe Diameter (mm)	Application	Additional Information
2009	Yang et al.	PAE	4.2	Rat gastrointestinal tract	—
2010	Yuan et al.	PAIE	10	Pig colorectal wall	360° view
2012	Yang et al.	PA/USE	3.8	Rat lower gastrointestinal tract and rabbit upper gastrointestinal tract	270° view
2012	Yang et al.	PA/USE	2.5	Adult Sprague-Dawley rat descending colon	Only suitable for lower GI tract
2014	Yang et al.	PAE	3.2	New Zealand rabbit upper esophagus	Flexible shaft-based
2015	Yang et al.	OR-PAEM	3.8	Rat colorectum	—
2018	Xiong et al.	AF-PAE	9	Rabbit rectum	360° view
2018	Li et al.	PA/USE	2.5	Sprague-Dawley rat colorectum	360° view
2019	Li et al.	PA/USE	1.5	Sprague-Dawley rat rectal wall	—
2019	Li et al.	OR-PAE	5	Porcine small intestine and rabbit rectum 3D vascular networks	Has a large-depth-of-field
2019	Xiong et al.	PAE	3	Rabbit colorectum	—
2022	Kim et al.	PA/USE	3.38	Esophagogastric junction of a swine and rat colorectum	—
2022	Pang et al.	PA/USE	—	Porcine small intestine	Based on the line-focused transducer
2022	Liang et al.	PAE	2	Rat rectum	—
2022	Xie et al.	PA/USE	11	Pig rectum’s inner and outer walls	360° view
2023	Jiang et al.	AR-PA/USE	8	Rabbit VX2 tumor model	Centimeter-scale deep penetration and 360° view

PAE, photoacoustic endoscopy; PAIE, photoacoustic imaging endoscope; PA/USE, acoustic resolution-photoacoustic/ultrasound endoscopy; OR-PAEM, optical resolution photoacoustic endoscopic microscopy; AF-PAE, autofocusing photoacoustic endoscope; OR-PAE, optical resolution photoacoustic endoscopy; AR-PA/USE, acoustic resolution-photoacoustic/ultrasound endoscopy; 3D, 3-dimensional; GI, gastrointestinal.

However, this single-mode device provided limited information and in-depth knowledge of the structure and functionality of biological tissues could only be obtained using multimodal PAE. In 2012, Yang et al. presented an endoscope with simultaneous photoacoustic and ultrasonic modes, with a probe smaller than those of the first generation.^[34]^ In a later experiment, 2 rabbit esophagi were scanned both *ex vivo* and *in vivo* by making use of this PA-US endoscope, with the successful acquisition of 3D-PA images displaying vascular and luminal anatomy.^[35]^ In 2012, the authors developed another dual-mode endoscopic probe with a smaller outer diameter of 2.5 mm. This device was subsequently used to image the descending colon of adult rats *in vivo.*^[36]^ A few years later, both Abran and colleagues’ and Kang and colleagues’ teams reported 3-modal instruments for US, PA, and fluorescence imaging.^[37,38]^ Additionally, the slow imaging rates (<10 frames per second) of existing PA-US endoscopes limited their clinical translation; hence, a combined PA-US endoscopic system that could capture up to 50 frames per second was designed in 2019. With a 1.5-mm outer diameter, it could fit into the accessory channel of a standard endoscope, permitting the visualization of the rectal wall vasculature and anatomical architecture in Sprague-Dawley rats.^[39]^

Spatial resolution has always been a concern in PAEs. However, the pursuit of a deeper penetration often leads to a decreased imaging resolution. Currently, acoustic resolution photoacoustic endoscopic microscopy (AR-PAEM) focuses on the received acoustic signal and laser beam at the same point for imaging; however, optical resolution photoacoustic endoscopic microscopy (OR-PAEM) uses a highly concentrated laser beam to replace the field illumination of AR-PAEM, resulting in a higher resolution.^[40]^ Yang and colleagues’ team, for instance, developed an OR-PAEM system and demonstrated the viability of PAT-based intravital microscopy for the first time by imaging the colorectum of rats and creating a 3D depiction of its vasculature.^[41]^ Moreover, Li et al. created a large depth-of-field optical-resolution PAE (OR-PAE) in 2019 that retained a mostly consistent transverse resolution while imaging targets at various depths, without requiring axial scanning. Its implementation demonstrated the ability to examine biological tissues at various depths and the potential of the endoscope for clinical colorectal applications.^[42]^

On this basis, various research groups have pursued significant improvements by refining the scanning systems in various ways. The most common method involved improving the mechanical scanning system. To improve the signal acquisition time, a fast preclinical PAI endoscope with coaxial characteristics of sound, ring, and light transducer arrays was developed by Yuan et al. in 2010, which could receive PA signals from a 360° field of view (FOV).^[43]^ In 2014, Yang et al. developed a novel catheter-based mechanical scanning PAE system using a flexible shaft; the outer diameter of the flexible catheter component was 3.2 mm, which allowed it to fit into the clinical video endoscope channel.^[44]^ In 2016, Xiao and colleagues’ team proposed a hollow structure and epoxy lens-focusing sensor for AR-PAEM.^[45]^ In gastrointestinal tract imaging, probes face the challenge of optical defocusing, which leads to a decrease in transverse resolution. To solve this issue, Xiong et al. developed an autofocusing PA endoscope (AF-PAE) in 2018 that achieved a focus-shifting range of 2 to 10 mm in a 360° FOV with high image contrast and transverse resolution.^[46]^ In 2018, Li et al. designed a PAE-EUS dual-mode device with a small enclosed imaging catheter with a 360° FOV. This catheter could be inserted into a 2.8-mm-diameter conventional endoscopic instrument channel. Using such system, the authors obtained *in vivo* 3D PA or US images of the colorectum of a rat.^[47]^ Moreover, Xiong et al. designed a new PAE for shape-adapting panoramic imaging in 2019. By applying a red-transparent water balloon, the probe could extract a holonomic vascular network layer and identify boundaries with improved axial resolution.^[48]^

In the past 2 years, mechanical scanning systems have been rapidly developed. A 3.38-mm-diameter catheter-based, combined optical-resolution PA-US microprobe system was developed by Kim et al. in 2022, for example, for obtaining PA and US images of a pig’s esophagogastric junction *in vivo*. Additionally, hierarchically developed mesh-like capillary networks have been visualized *in vivo* in the colorectum of mice.^[49]^ In the same year, Pang et al. reported an AR-PA/USE with a line-focused transducer that achieved autofocusing, thereby improving image contrast and signal-to-noise ratio (SNR) and solving the target signal difference flaw in the telephoto region of US imaging.^[50]^ In 2022, Xie et al. designed a compact PAE/EUS probe that provided 360° structural and functional imaging, using a ring-shaped laser beam and circular array transducer to avoid the instability caused by point-by-point mechanical scanning. The imaging distance determined by phantom experiments was large enough to span the range of 12 to 30 mm, which is consistent with the complex intestinal environment, and the probe could access functional information at the inner surface and at various depths.^[51]^ In 2023, Jiang et al. developed a low-cost AR-PA/USE system with cross-scale imaging capability, multispectral imaging, centimeter-scale deep penetration, and a 360° FOV. This system could detect tumor angiogenesis and accurately visualize the depth of tumor invasion using indocyanine green (ICG).^[52]^

Compared with the mechanical scanning system, the optical scanning–based PAE system is more compact and has a better SNR and faster scanning speed. In 2017, Guo and colleagues, for instance, reported a high-resolution PA endomicroscopy probe that utilized microelectromechanical systems scanning; resected rectums and *in vivo* mouse ears were photographed to demonstrate the potential of the probe in biological and therapeutic applications.^[53]^ Furthermore, a miniature endoscope using a novel Fabry-Pérot acoustic transducer was reported in 2018.^[54]^ In 2019, Chen and colleagues designed a mini-PAE using a Fabry-Pérot acoustic transducer, with a 2.4-mm-diameter, high-lateral-resolution, and wide FOV.^[55]^ In 2022, Liang and colleagues developed a 2-mm-diameter optical-resolution PAE utilizing the optical heterodyne detection of US, which could image gastrointestinal hemodynamics *in vivo*. This provided a spatial resolution of 7.4 μm, enabling visualization of the change in tissue oxygen saturation (spo_2_) during rectal inflammation and providing the related metabolic data.^[56]^

**Figure 1:**
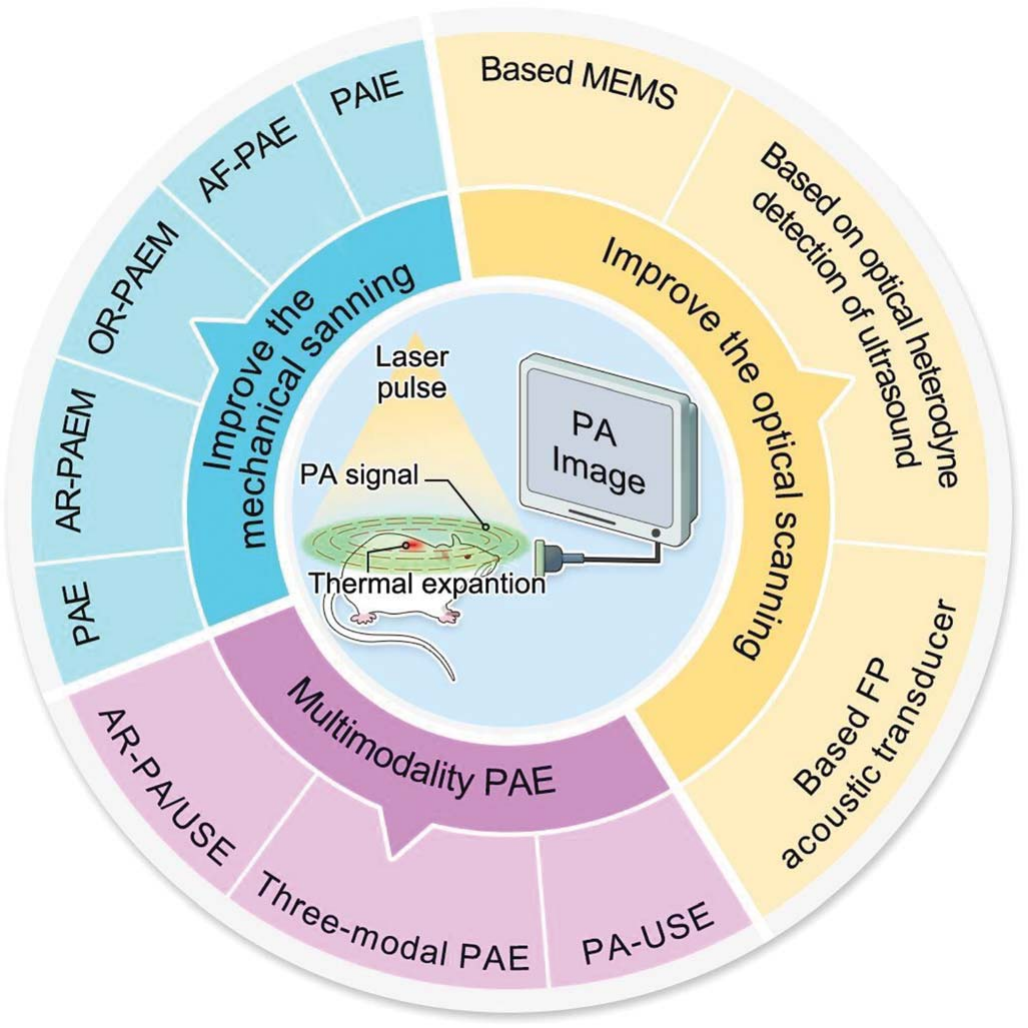
The development of PAE. PAE, photoacoustic endoscopy; AR-PAEM, acoustic resolution photoacoustic endoscopic microscopy; OR-PAEM, optical resolution photoacoustic endoscopic microscopy; AF-PAE, autofocusing photoacoustic endoscope; PAIE, photoacoustic imaging endoscope; MEMS, microelectromechanical systems; FP, Fabry-Pérot; PA-USE, photoacoustic-ultrasound endoscopy; AR-PA/USE, acoustic resolution-photoacoustic/ultrasound endoscopy; PA, photoacoustic.

**Figure 2:**
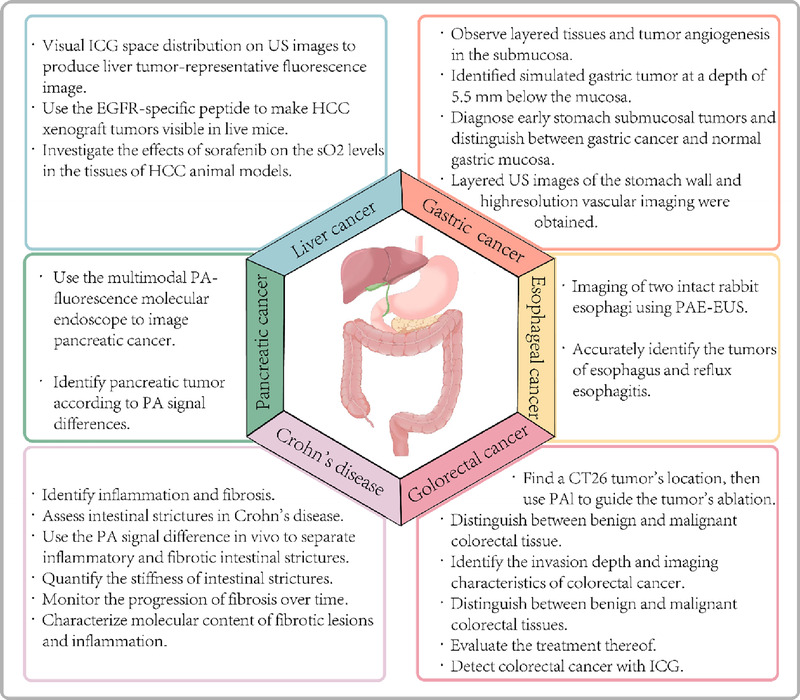
Applications of photoacoustic imaging in digestive diseases. ICG, indocyanine green; US, ultrasound; EGFR, epidermal growth factor receptor; HCC, hepatocellular carcinoma; so_2_, oxygen saturation; PA, photoacoustic; US, ultrasound; PAE-EUS, photoacoustic endoscopy-EUS; PAI, photoacoustic imaging.

In addition to the advances in PAE devices, contrast agents have been developed to improve the sensitivity and specificity of PAE.^[57]^ Using PA contrast agents, tissue function and the related molecular information can be better acquired with PAE, thereby aiding in the subsequent evaluation of tissue structure, shape, physiological, and metabolic functions, as well as pathological features of digestive tract tumors. Endogenous contrast agents are molecular substances, such as the most commonly used hemoglobin. Measuring hemoglobin levels can help identify differences in abnormal blood vessels, hypoxia, and oxygenation between tissues to detect and characterize cancerous tissue^[8–10,58];^ hence, this can be additionally used for perfusion and inflammation imaging.^[59]^ Other endogenous contrast agents include melanin,^[61,62]^ lipids,^[63]^ water,^[64]^ and bilirubin.^[65]^ Exogenous contrast agents have been introduced for drug delivery, cancer detection, and therapeutic diagnostics using nanotechnology for photothermal therapy^[59]^ and include small organic molecules,^[66,67]^ nanoparticles,^[68–74]^ and carbon-based contrast agents^[75]^ (Figures 1 and 2).

## Applications in benign and malignant diseases of the digestive system

### Applications in benign diseases

#### Crohn disease

Traditional noninvasive imaging cannot distinguish fibrosis from inflammation in strictures. In contrast, PAI can assess the hemoglobin and collagen levels in tissues to identify intestinal stenosis.

In 2016, PAI was used to detect inflammation and fibrosis in Crohn disease by simultaneously measuring the amounts of collagen and hemoglobin in animal and human tissues.^[76]^ Furthermore, Zhu et al. utilized a PA-US dual-mode device to distinguish between inflammatory and fibrotic intestinal strictures in 2018^[77]^ and found that the different strictures showed substantial changes in the PA signal intensities.^[78]^ In 2019, the authors discovered that concurrent PA-US imaging could quantify the stiffness of intestinal strictures.^[79]^ Zhu et al. designed a prototype capsule-shaped AR-PA endoscope, which discriminated fibrosis and intestinal inflammation in rabbit models *in vivo*; PAI was able to follow the progression of intestinal fibrosis, thereby providing crucial data for therapeutic decision-making.^[80]^ In 2022, Zhu et al. demonstrated that a prototype endoscopic PAT and US balloon catheter could identify the molecular structure of colonic fibrotic lesions and inflammation *in vivo* in rabbit models^[81]^ (Table 2).

**Table 2:** PAE applications in digestive diseases.

Disease	Year	Content of Research
Crohn disease	2016	Identify inflammation and fibrosis. Assess intestinal strictures in Crohn disease.
	2018	Use the PA signal difference *in vivo* to separate inflammatory and fibrotic intestinal strictures.
	2019	Quantify the stiffness of intestinal strictures.
	2019	Monitor the progression of fibrosis over time.
	2022	Characterize molecular content of fibrotic lesions and inflammation.
Esophageal cancer	2015	Imaging of 2 intact rabbit esophagi using PAE-EUS.
	2017	Accurately identify the tumors of esophagus and reflux esophagitis.
Gastric cancer	2018	Observe layered tissues and tumor angiogenesis in the submucosa.
	2018	Assess the feasibility of detection in gastric cancer.
	2019	Identified simulated gastric tumor at a depth of 5.5 mm below the mucosa.
	2020	Diagnose early stomach submucosal tumors and distinguish between gastric cancer and normal gastric mucosa.
	2021	Layered US images of the stomach wall and high-resolution vascular imaging were obtained.
Liver cancer	2014	Visual ICG space distribution on US images to produce liver tumor-representative fluorescence image.
	2016	Use the EGFR-specific peptide to make HCC xenograft tumors visible in live mice.
	2018	Investigate the effects of sorafenib on the spo_2_ levels in the tissues of HCC animal models.
Pancreatic cancer	2017	Use the multimodal PA-fluorescence molecular endoscope to image pancreatic cancer.
	2018	Identify pancreatic tumor according to PA signal differences.
Colorectal cancer	2010	Distinguish human healthy tissue and colorectal cancer tissue.
	2010	Find a CT26 tumor’s location, then use PAI to guide the tumor’s ablation.
	2018	Distinguish benign and malignant colorectal tissue. Identify the invasion depth and imaging characteristics of colorectal cancer.
	2019	Distinguish benign and malignant colorectal tissues. Evaluate the treatment thereof.
	2023	Detect colorectal cancer with ICG.

PA, photoacoustic; PAE-EUS, photoacoustic endoscopy-EUS; US, ultrasound; ICG, indocyanine green; EGFR, epidermal growth factor receptor; HCC, hepatocellular carcinoma; spo_2_, oxygen saturation; PAI, photoacoustic imaging.

### Applications in malignant diseases

#### Esophageal cancer

In 2015, Yang et al. imaged 2 intact rabbit esophagi using PA-US endoscopy and the first PA images of the vasculature of the esophagus of a vertebrate; this suggested that PAE may be a useful tool for evaluating the esophageal function and structure.^[35]^ In 2017, an *in situ* morphological and biomechanical analysis of reflux esophagitis and esophageal tumors in rabbit esophagi using PAE demonstrated that this technique may be able to detect early esophageal diseases in clinical settings.^[82]^

#### Gastric cancer

Although gastroscopy can only diagnose superficial gastric cancer, EUS can assess both the mucosa and submucosa to determine the tumor stage and invasion extent yet cannot demarcate the boundaries with the surrounding blood or lymphatic vessels. PAI overcomes these limitations and may be used for the clinical diagnosis of early gastric cancer (EGC), providing knowledge on invasion depth, morphological characteristics, and other structural information, thereby reducing the rate of missed EGC diagnoses.

In 2018, Wang et al. introduced an AR-PAM system, which obtained PA images of the mucosa, submucosa, and blood vessels beneath the mucosa, suggesting that PAI combined with endoscopy can detect tumor angiogenesis and layered tissues in the submucosa.^[83]^

In 2019, Wu et al. used a PA scanning imaging system to identify submucosal EGC, distinguishing the simulated gastric tumor at a depth of 5.5 mm below the mucosa.^[84]^

In 2020, a real-time PA-US dual-mode device was used to diagnose submucosal EGC. Additionally, the system could quantitatively characterize the gastric wall structure and tumor morphology, along with the tumor location and boundaries.

In 2021, Kim et al. obtained layered US and high-resolution vascular images of the stomach walls of large animals for the first time, as well as mucosal vascular PA images with a 1.90-mm depth. Such techniques are expected to have therapeutic applications by aiding in distinguishing T-indicators, diagnosing EGC, and assisting in endoscopic submucosal dissection.^[85]^

#### Liver and pancreatic cancer

With the rapid development of PAI technology, its high resolution and contrast contribute to improve the efficiency of liver cancer diagnosis and provide a basis for the accurate location of focal boundaries and define treatment.

In 2014, Miyata et al. used intraoperative fluorescence and US imaging to show the potential of PAI with ICG in improving hepatic tumor localization and differential diagnosis,^[67]^

.In 2016, Zhou et al. visualized hepatocellular carcinoma (HCC) xenograft tumors with epidermal growth factor receptor–specific peptides in live mice using PAI, showing that the method may improve the detection rate of liver cancer and metastasis.^[86]^

Sorafenib is a first-line therapy for advanced HCC and can affect the oxygenation state of tumors. Therefore, monitoring the spo_2_ levels in HCC after sorafenib treatment is necessary. In 2018, Lee et al. showed that spo_2_ measured using PAI might serve as a marker for noninvasive monitoring of the therapeutic response in orthotopic HCC models in animals.^[87]^

Moreover, PAI has several advantages in the diagnosis of pancreatic cancer. Dai et al. performed multimodal PA fluorescence molecular endoscopic imaging in a rat pancreatic cancer model.^[88]^ Studies using specific fluorescent probes combined with PAI have successfully identified tumors and showed that the average PA signal of pancreatic tumor sites is 3.7 times higher than that of the surrounding tissues.^[89]^

Although endoscopic US is one of the best diagnostic modalities for pancreatic diseases, it cannot provide information on blood supply, fat, or oxygen contents.^[90–94]^ US-PA multimode endoscopy, on the other hand, provides a more accurate diagnosis of liver and pancreatic cancers. Furthermore, PAI technology has been used to locate lymph nodes, determine lymphatic vessel morphology, and achieve sentinel lymph node biopsy.^[5,95]^ This suggests that PAE may be used to perform real-time puncture biopsies in the future.

#### Colorectal cancer

Colorectal cancer is one of the most fatal malignancies. Consequently, accurate imaging methods to evaluate its staging and therapeutic response are urgently required.

In 2010, PA-based reconstruction of the colorectal wall of a pig was performed *in vitro* using preclinical PAI endoscope. Additionally, this system effectively distinguished *ex vivo* colorectal cancer tissues from healthy human colorectal tissue.^[43,96]^

Cui et al. used PAI to locate and ablate a CT26 tumor subcutaneously implanted in the hips of a BALB/c mouse in 2010.^[97]^

In 2018, Leng and colleagues used a novel coregistered US and AR-PAM system to image 8 colorectal specimens; this helped recognize both the colorectal cancer imaging characteristics and the degree of invasion.^[96]^

Yang et al. used a real-time coregistered PAT/US system to characterize and image 32 human rectal and colon samples *ex vivo* in 2019. The PA signal surrounding the malignant tumors, especially on the mucosal surface, was significantly increased, whereas that inside the tumor was decreased, suggesting that PAT may be able to differentiate cancerous and healthy tissues in the rectum and colon.^[98]^

In 2023, Jiang et al. showed that AR-PA/USE was effective for colorectal cancer detection if used with a correct contrast agent, such as ICG.^[52]^

PAI or PA multimodal imaging provides high-resolution functional imaging that can be used as a novel auxiliary tool for localizing and characterizing colorectal tumors. PAE can also be theoretically used for *in vivo* imaging to distinguish benign and malignant colorectal tissues; thus, this method is a potential new instrument for the early diagnosis of colorectal cancer.

## Conclusion and future prospects

The field of PAI has rapidly grown in recent years. In particular, PAE and multimodality PAE are promising tools for assessing internal organs and diagnosing vascular disorders. Precise guided biopsy and treatment may be a potential direction for future development. Although substantial progress has been made in PAE, obstacles to its implementation in clinical settings remain. First, several clinical PAE studies are still in the feasibility-testing phase, and large-scale clinical trials are required. Second, an unformed system and its high cost impede the progress of clinical applications. Furthermore, ensuring high-SNR imaging requires creating a small flexible probe that can enter the digestive endoscopic instrument channel with a full 360° FOV. Ongoing research will enable the development of a PAE imaging technology suitable for the digestive tract, which can achieve a full FOV and is compatible with digestive endoscope instrument channels. Additionally, key parameters closely related to tumor growth and development can be comprehensively obtained to improve the diagnosis of benign and malignant tumors and their accurate staging. We believe that ongoing innovation and optimization will allow PAE technology to become an indispensable clinical tool for the diagnosis and treatment of various gastrointestinal diseases.

## Acknowledgments

None.

## Source of Funding

This study was funded by the Scientific Research Fund of Liaoning Province Education Department, grant no. JYTQN 2023025; and the Natural Science Foundation of Liaoning Province, grant no. 2024-MS-075.

## Ethical Approval

Not applicable.

## Informed Consent

Not appliable.

## Conflicts of Interest

Siyu Sun is Editor-in-Chief of the journal, and Zhendong Jin is an Associate Editor. The other authors declare that they have no financial conflict of interest. The article was subject to the journal’s standard procedures, with peer review handled independently of this editor and his research groups.

## Author Contributions

Ideation and planning of the work was done by Kai Zhang and Lufan Shen. Data collection and analysis were performed and the first draft of the manuscript was written by Lufan Shen. Kai Zhang and Nan Ge revised and supplemented the paper. Tianan Jiang, Zhendong Jin, and Siyu Sun provided valuable comments on the conduct of the work. All authors read and approved the final manuscript.

## Data Availability Statement

No additional data is available.
